# Supplementary material for: Synergistic Halide- and Ligand-Exchanges of All-Inorganic Perovskite Nanocrystals for Near-Unity and Spectrally Stable Red Emission
Source: Nanomaterials (Basel). 2023 Aug 14;13(16):2337. doi: 10.3390/nano13162337 (PMC10458086; doi:10.3390/nano13162337)
Supplement: Supplementary file 1 [file nanomaterials-13-02337-s001.zip › nanomaterials-2525402-supplementary.pdf]

Supporting Information

Synergistic halide- and ligand-exchanges of all-inorganic perovskite nanocrystals for near-unity and spectrally stable red emission

Kaiwang Chen[1], Dengliang Zhang[1], Qing Du[1], Wei Hong[1], Yue Liang[1], Xingxing Duan[1], Shangwei Feng[1], Linfeng Lan[1], Lei Wang[2], Jiangshan Chen[1,*], Dongge Ma[1,*]

1 Institute of Polymer Optoelectronic Materials and Devices, State Key Laboratory of Luminescent Materials and Devices, Guangdong Provincial Key Laboratory of Luminescence from Molecular Aggregates, South China University of Technology, Guangzhou 510640, China; chenkw1009@163.com (K.C.); msdlzhang@foxmail.com (D.Z.); duqing326@126.com (Q.D.); yc37821@umac.mo (W.H.); 18335743033@163.com (Y.L.); 202110183327@scut.edu.cn (X.D.); 13060657551@163.com (S.F.); lanlinfeng@scut.edu.cn (L.L.)

2 Wuhan National Laboratory for Optoelectronics, Huazhong University of Science and Technology, Wuhan 430074, China; wanglei@mail.hust.edu.cn

* Correspondence: msjschen@scut.edu.cn (J.C.); msdgma@scut.edu.cn (D.M.)

Characterization

The measurements of PL spectra, PLQY and spectral stability were performed with a commercialized measurement system of PL-MS(III) from Guangzhou BiaoQi Optoelectronics Technology Development Co., Ltd., and a 365-nm LED was used as the excitation light source. The ultraviolet-visible absorption spectra were measured with a HP 8453 spectrophotometer. The time-resolved photoluminescence (TRPL) decay curves were measured by Edinburgh FLS980 with a 375 nm laser. The X-ray diffraction (XRD) measurements were performed with a Rigaku Smart lab (3 kW) XRD patterns with Bragg-Brentano focusing, a diffracted beam monochromator and a conventional Cu target X-ray tube set to 40 kV and 30 mA. The X-ray energy of 10 kV, a sample-to-detector distance of 313 mm, and an incident angle of 0.30° were selected for the experiment. The film samples for XRD measurements were prepared by spin-coating perovskite NCs on glass substrates. The X-ray photoelectron spectroscopy (XPS) measurements were conducted by using an Axis Supra+ spectrometer with a monochromatic Al KR radiation source (1486.6 eV). The XPS samples were prepared by dropping the purified NCs inks onto glass substrates. The Fourier transform infrared (FTIR) spectra were measured with a Bruker Vector 33 spectrometer. The FTIR samples were prepared by drying the purified NCs and pressing their powders with KBr. High-resolution transmission electron microscopy (HRTEM) images were taken on a JEOL JEM-2100F TEM instrument operated at an acceleration voltage of 200 keV. The broadband femtosecond TA spectra of the perovskite solutions were taken using the Ultrafast System HARPIA TA spectrometer. A Yb: KGW amplifier (PHAROS, Light Conversion) supplied laser beams centered at 1030 nm with pulse duration of ~100 fs, pulse repetition rate of 40 kHz, and a maximum pulse energy of 100 μJ. The output of the amplifier was split into two streams of pulses. One was used to drive an optical parametric amplifier (ORPHEUS, Light Conversion) to obtain the pump beam. The residual stream was

directed into an ultrafast spectroscopic system (HARPIA-TA, Light Conversion) to generate the white light continuum probe beam. In the spectrometer, the pump chopped at the frequency of 20 kHz was spatially and temporally overlapped with the probe beam on the sample. The pump beam was focused onto the sample with a beam size of 300 μm, which overlapped with the smaller-diameter (200 μm) probe beam. The pump and probe pulses were crossed on the sample and the transmitted probe beam was recorded using a CCD linear Si detector coupled to a monochromator. The perovskite solutions were sealed in the quartz cuvettes. The WLEDs were powered by Keithley 2400 Sourcemeter and their spectra were recorded with a spectrometer (QE65 Pro, Ocean Optics).

Computational methods

Electronic structure calculations were performed with the density functional theory as implemented in the Vienna ab initio simulation package, employing the projected augmented wave potentials to describe the atomic core electrons and the plane wave basis set to expand the Kohn–Sham electronic states. For the exchange and correlation functional, the generalized gradient approximation (GGA) in the Perdew–Burke–Ernzerhof (PBE) format was used. The kinetic energy cutoff was set to 400 eV for all calculations in this work.

The Brillouin zone was sampled by a (2×2×2) k-points mesh with Gamma point centered. The convergence criterions of energy and force calculations were set to 10^{-5} eV/atom and 0.01 eV $Å^{-1}$, respectively. The 2×2 supercell of $CsPbBr_3$ and $CsPbI_3$ model were constructed, respectively. The doping formation energy was defined as:

$$E_f = E_{doping} - E_{perfect} + \mu_{Zn} - \mu_{Pb}$$

where E_f is the doping formation energy, Edoping are the total energy of $CsZn_{0.125}Pb_{0.875}Br_3$ and $CsZn_{0.125}Pb_{0.875}I_3$, respectively, Eperfect are the total energy of $CsPbBr_3$ and $CsPbI_3$, respectively, μ_{Zn} represents the chemical potential of Zn atom, μ_{Pb} represents the chemical potential of Pb atom.

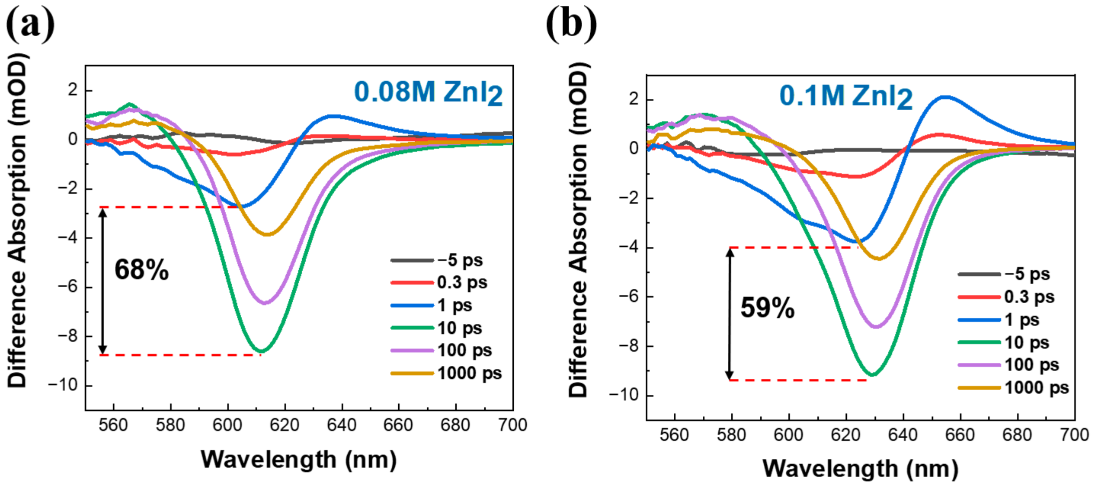

Figure S1. TA features for (a) 0.08M ZnI_2 NCs and (b) 0.1M ZnI_2 NCs.

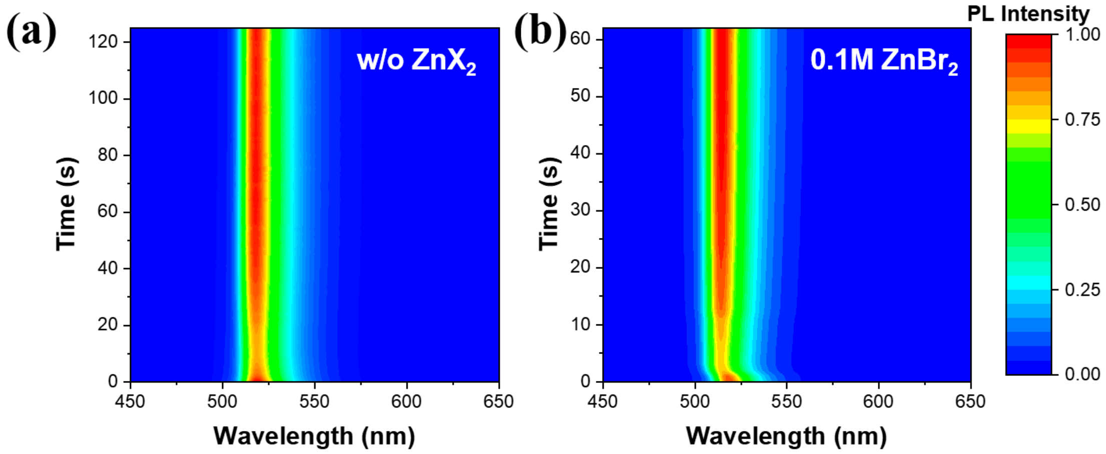

Figure S2. The fitted 2D PL spectra of the treated perovskite NCs over time, with w/o M ZnX_2 (0-120s) (a), with 0.1 M $ZnBr_2$ (0-60s) (b). Color scale bar is provided on the right side for the intensity of the PL.

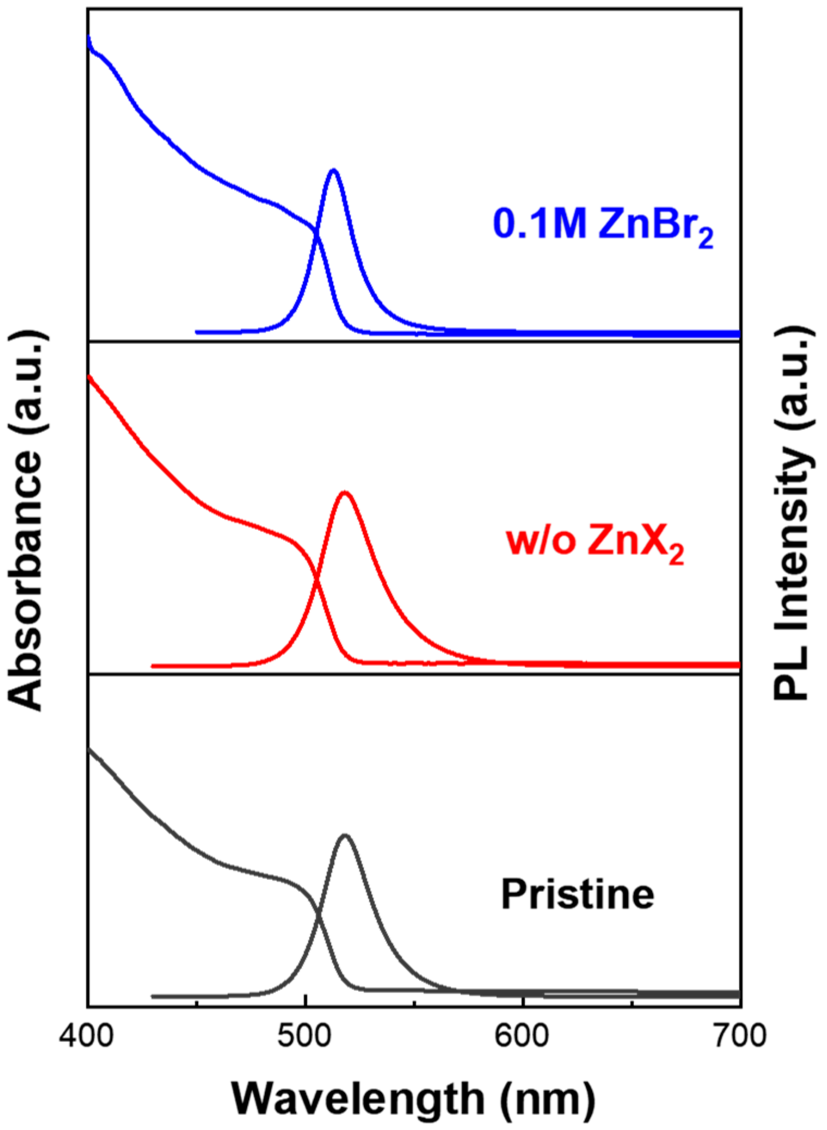

Figure S3. UV-vis absorption and PL spectra of the pristine $CsPbBr_3$ NCs and the exchanged $CsPbBr_3$ NCs treated with precursor solutions of without Zn^{2+} (w/o ZnX_2) and with $ZnBr_2$ (0.1M $ZnBr_2$).

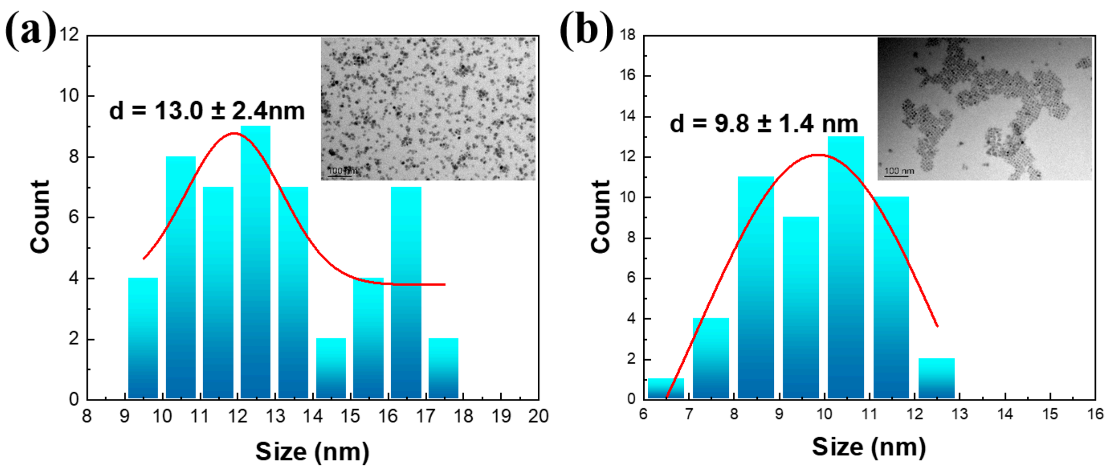

Figure S4. Size histogram of the exchanged $CsPbBr_3$ NCs: (a) w/o ZnX_2 and (b) 0.1M $ZnBr_2$ (inset: TEM images of the perovskite NCs).

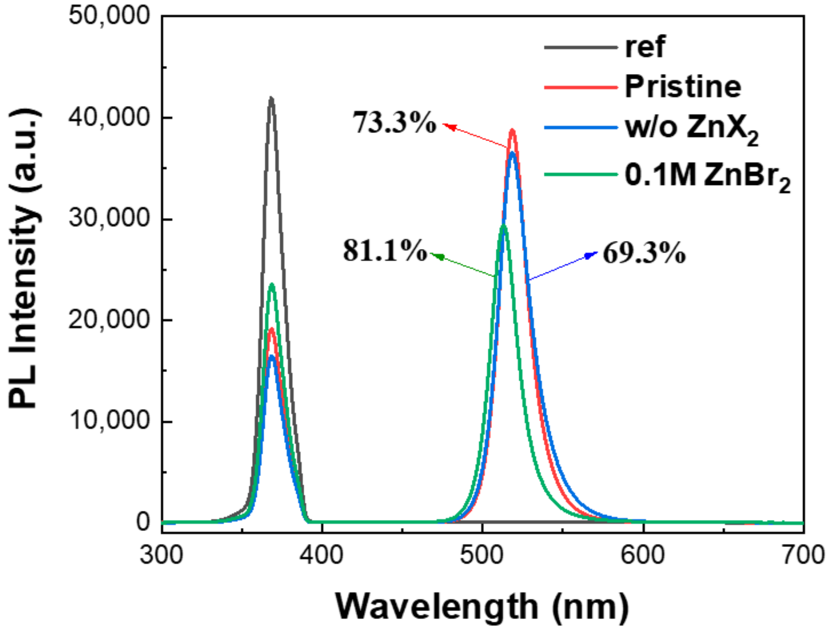

Figure S5. PLQY spectra of the pristine $CsPbBr_3$ NCs and the exchanged $CsPbBr_3$ NCs of w/o ZnX_2 and 0.1M $ZnBr_2$ in solutions.

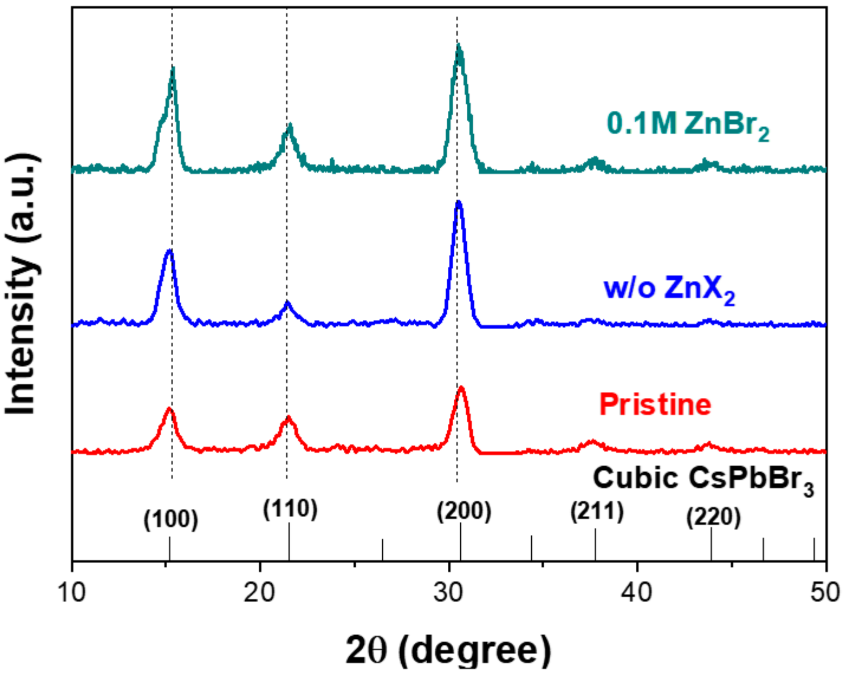

Figure S6. XRD spectra of the films of the pristine $CsPbBr_3$ and the exchanged $CsPbBr_3$ NCs of w/o ZnX_2 and 0.1M $ZnBr_2$.

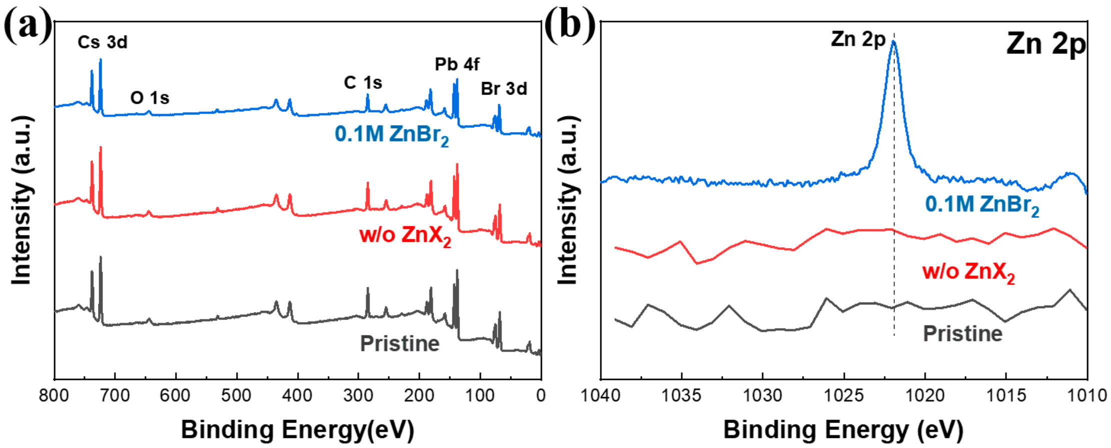

Figure S7. XPS spectra of the perovskite NCs powders of the pristine $CsPbBr_3$ and the exchanged $CsPbBr_3$ NCs of w/o ZnX_2 and 0.1M $ZnBr_2$: survey spectra (a), Zn 2p spectra (b).

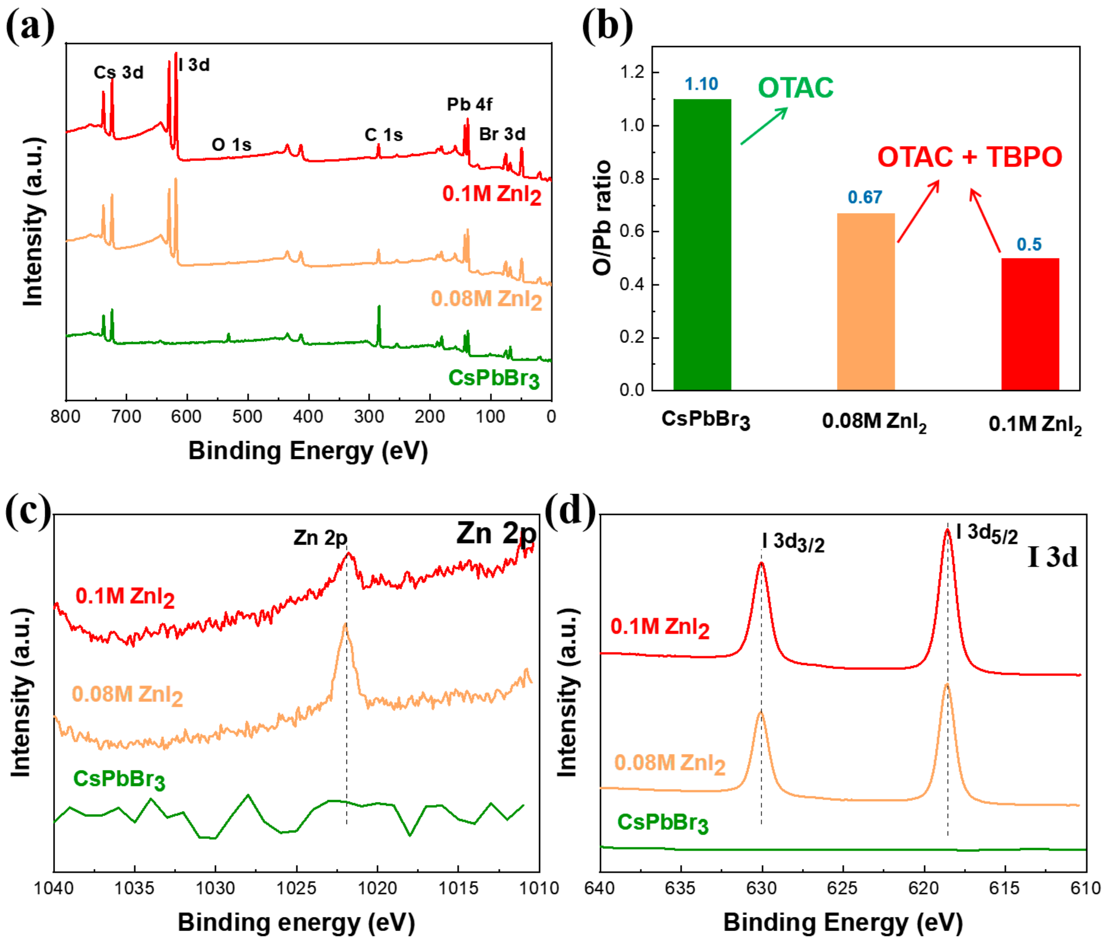

Figure S8. XPS spectra of perovskite NCs powders: survey spectra (a), N/Pb atomic ratios in the perovskite NCs calculated from the XPS data (b), Zn 2p spectra (c), I 3d spectra (d).

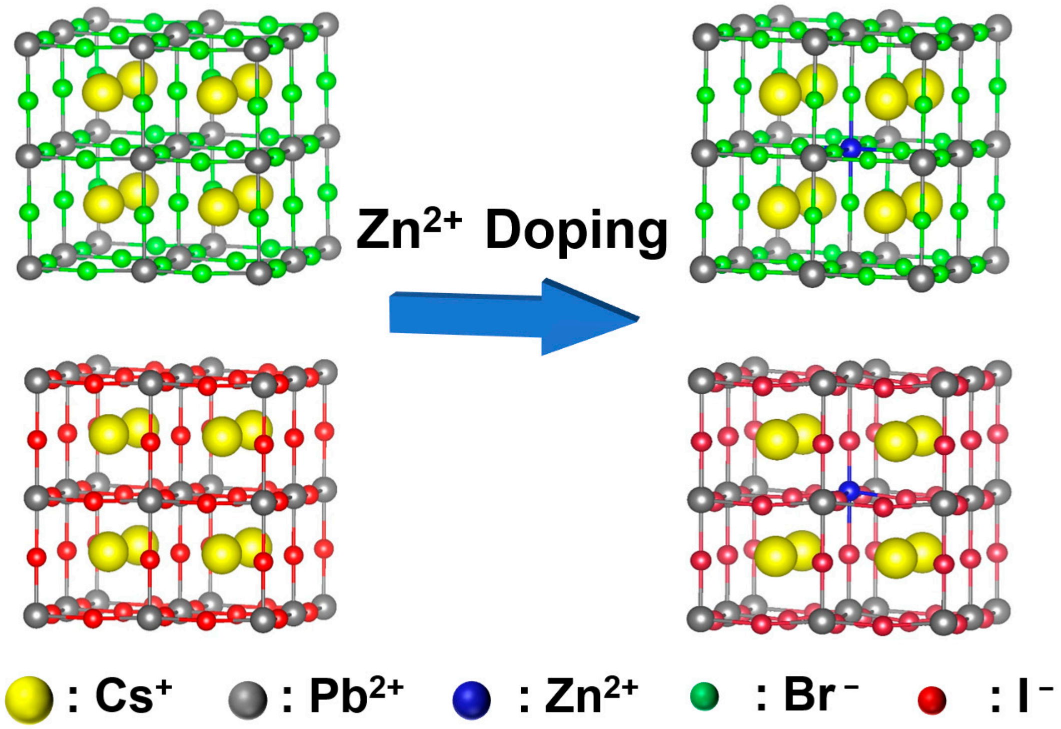

Figure S9. Schematic of the crystal structure of $CsPbBr_3$ and $CsPbI_3$ NCs before and after Zn^{2+} doping.

Table S1. Summary of the optical properties of the different NCs.

ZnI_2	0	0.04M	0.06M	0.08M	0.1M
**PLQY (%)**	72.75	21.15	41.01	85.02	~100
**PL (nm)**	518	550	584	619	640
**FWHM (nm)**	20	23	28	32	33

Table S2. Fitted PL lifetimes of different NCs solution measured by using the bi-exponential decay: $I(t) = A_1\exp(-t/\tau_1) + A_2\exp(-t/\tau_2)$.

	**0.08M ZnI_2**	**0.1M ZnI_2**
A_1	0.27	0.21
τ_1 (ns)	16.30	16.42
A_2	0.73	0.79
τ_2 (ns)	71.75	82.36
χ^2	0.996	0.998
τ_{avg} (ns)	56.62	68.73

Table S3. The calculated formation energies (△E) of the Zn-doping perovskite NCs based on the crystal structure as shown in Figure S9.

Perovskite	$CsZn_{0.125}Pb_{0.875}Br_3$	$CsZn_{0.125}Pb_{0.875}I_3$
△E (eV)	0.80<br>(heat absorption)	0.79<br>(heat absorption)
